# A Clinical Picture of the Visual Outcome in Adamantiades-Behçet's Disease

**DOI:** 10.1155/2015/120519

**Published:** 2015-10-19

**Authors:** Michele Figus, Chiara Posarelli, Timothy G. Albert, Rosaria Talarico, Marco Nardi

**Affiliations:** ^1^Department of Surgical, Medical, Molecular Pathology and Department of Emergency, University of Pisa, 56100 Pisa, Italy; ^2^Rheumatology Unit, Department of Clinical and Experimental Medicine, University of Pisa, 56100 Pisa, Italy

## Abstract

Adamantiades-Behçet's disease is a multisystemic vasculitis with multiorgan involvement. Ocular disorders occur often in this syndrome typically in the form of a relapsing-remitting panuveitis and vasculitis and can lead to blindness as one of its most disabling complications if left untreated. There are known risk factors related with the worst visual prognosis, which require early and intensive treatment in order to obtain a rapid suppression of inflammation and to prevent future relapses. The management strategy to avoid vision loss and blindness currently involves the use of local and systemic drugs including steroids and immunosuppressive and biologic agents. This review aims to demonstrate how the introduction and the use of biologic agents improves the visual outcome of patients with Adamantiades-Behçet's disease.

## 1. Introduction

Adamantiades-Behçet's disease (ABD) is a chronic, multisystemic disorder characterized by recurrent inflammation that involves multiple organ systems throughout the body. It has a high prevalence along the ancient “Silk Road,” but it is an important cause of morbidity throughout the world. The underlying pathology in ABD is a vasculitis that affects both the arteries and the veins in all organ systems. The involvement of major organs can cause permanent damage and severe complications that may be even life threatening [1]. Ocular involvement is present in around half of ABD patients with the percentage varying among 70% in young men with ABD and 30% in women and elderly patients [1–3]. Ocular manifestations usually manifest themselves within 5 years from the onset of the disease [2]. Further, bilateral involvement is frequent and is reported in 75–80% of ABD patients [2].

The ophthalmic findings described in ABD can involve either the anterior, posterior, or both segments of the eye and can be classified as suggested in the review by Ozyazgan et al. [4] as “reversible changes” or “irreversible changes.” The reversible changes appear during the activation and completely disappear after the deactivation of disease; the irreversible changes develop slowly during the course of inflammation and do not disappear after remittance. The most sight-threatening complications often are consequences of both the reversible and the irreversible modifications to the anterior or the posterior segment of the eye. Complicated cataract, macular oedema, secondary glaucoma, epiretinal membrane, macular hole, and optic disc atrophy may cause vision loss and, if not treated, also blindness. The risk of blindness increases progressively reaching 25% at 10 years and remains constant thereafter [1]. Conventional treatment consists of prednisone, cyclosporine, azathioprine, and other immunosuppressive agents such as methotrexate and cyclophosphamide. Steroids are used usually for the rapid suppression of the inflammation but are quickly tapered to reduce the risks of secondary cataract and glaucoma. In patients with severe ocular involvement with vasculitis and relapses, immunosuppressive agents should be added to ameliorate the visual prognosis. Nonresponsive patients can also benefit from biologic agents. Interferon-alpha (INF-alpha), tumor necrosis factor-alpha (TNF-alpha) antagonists, and recently interleukin-1 (IL-1) blocking agents have been used with a significant improvement of visual acuity.

## 2. Steroid Treatment and Visual Outcome

In the early 1960s, the treatment of ocular manifestations of ABD was more dependent upon rheumatologist-prescribed corticosteroid therapy for extraocular manifestations of this disease, while corticosteroid monotherapy was the mainstay of treatment [5]. Currently if the inflammation is located predominantly in the anterior segment, topical treatment modalities are recommended together with mydriasis. Dexamethasone 0.1%, prednisolone 1%, and fluorometholone 0.1% have been employed topically or through subconjunctival injection (methylprednisolone acetate 20 mg) in severe anterior segment inflammation and for treating hypopyon [2]. Systemic steroid regime is necessary in case of posterior segment involvement. Initially, patients are treated with oral prednisone 1 to 2 mg/kg/daily for four days with gradual tapering of the dose according to the clinical signs [6], or with high-dose intravenous methylprednisolone [7]. Looking at a study of the National Eye Institute, comparing three decades of treatment [8], mean visual acuity was significantly worse in the 1960s than in the following decades, and accordingly the mean logarithm of the minimum angle of resolution (logMAR) score decreased with each decade: respectively, 0.91 logMAR in the 1960s, 0.82 logMAR in the 1980s, and 0.46 logMAR in the 1990s. This could be explained by the fact that the use of steroids as monotherapy fell significantly from the 1960s (96%) compared to the 1980s (8%) and the 1990s (16%) (*P* < 0.001). In the 1970s, it was reported that vision was lost after an average of 3.36 years from the onset of visual symptoms [9]. Mishima and associates found that more than 50% of the Japanese patients with ABD had a visual acuity of 0.1 decimal or less in 5 years [10].

## 3. Immunosuppressive Agents and Visual Outcome

### 3.1. Cyclosporine A

Cyclosporine A (CSA) is an 11-amino acid cyclic peptide. It is an alkylating agent that appears to affect preferentially immunocompetent T-lymphocytes [11]. CSA, in a dose of 5 mg/kg/day, was found to be effective in arresting the inflammatory activity in the eye of patients with Behçet's disease, resulting in a rapid improvement in visual acuity. The response rate to CSA in ABD patients varied between 80 and 91% [12–14]. In a first report published in 1987, visual acuity improved in 12 eyes, was unchanged in three eyes, and worsened in one eye of patients treated with CSA [12]. Ozyazgan in a single masked trial demonstrated that there was an initial improvement in visual acuity with 5 mg/kg/day of CSA versus monthly 1 gram of intravenous bolus of cyclophosphamide. However, this improvement disappeared during the follow-up time, and at the end of 24 months of observation, visual acuity remained approximately the same in both groups.

In the cyclophosphamide group, no significant change in visual acuity occurred; for this reason the author suggested CSA for short-term use [13].

According to a previous report, Masuda has demonstrated in a double masked trial that CSA 10 mg/kg per day was effective in treating ocular manifestation of Behçet's disease and he also observed that the efficacy did not weaken in long-term treatment [14, 15]. In these patients treated with CSA, caution is required regarding the potential for development of hypertension and renal failure.

### 3.2. Azathioprine

Azathioprine (AZA) is a purine nucleoside analogue. Immunologically, it decreases the number of peripheral T and B lymphocytes and further reduces mixed lymphocytes reactivity, interleukin-2 synthesis, and IgM production [11]. The azathioprine randomized controlled trial showed that AZA 2.5 mg/kg per day was superior to a placebo in preserving visual acuity in patients with established eye disease, but there was no evidence that AZA was useful in restoring compromised vision. It has been suggested also that AZA can protect against the development of second-eye disease [16]. According to the previous reports, another randomized controlled double blind study demonstrated that blindness and a 2-line drop in the visual acuity of the right eye occurred significantly more frequently among the patients originally allocated to the placebo group compared with patients who originally received AZA, despite posttrial treatment for patients in both groups when needed [17]. Tugal-Tutkun I described a cohort of 36 childhood-onset uveitides, treated with oral corticosteroids and immunosuppressive treatment if necessary. Twelve patients received AZA as initial line drugs; looking at final visual acuity, 50% of the patients showed a visual acuity of 0.6 decimals or better, but 22.7% had a visual acuity of 0.1 decimals and six patients (16.6%) were legally blind at the time of last visit [18]. In patients without eye involvement, the use of AZA seems to prevent the development of new eye disease even during follow-up and compared with the placebo group blindness occurred only in 13% of patients, which still remained a high percentage though [17]. Concomitant use of azathioprine and/or cyclosporine may improve the outcome.

### 3.3. Methotrexate

Methotrexate (MTX) is a folic acid analogue and an inhibitor of dihydrofolate reductase, the enzyme responsible for the conversion of dihydrofolate reductase in tetrahydrofolate reductase, essential for DNA replication [11]. Davatchi presented the results of a longitudinal study of up to 15 years, on 682 patients (5447 eye-years of follow-up) with Behçet's disease and ocular involvement. Patients were treated with MTX started at 7.5–15 mg/week and prednisolone was added at 0.5 mg/kg/daily and then adjusted as needed. At the end of the study, the visual acuity was improved in 46.5% of the eyes (20% recovered normal vision, 15,3% had useful vision and improved with the treatment, 2,4% were blind and recovered some vision, and 8,4% recovered at least one eye). In eyes with posterior uveitis, improvement was achieved in 75.4% of the eyes and in 53.7% of eyes with retinal vasculitis [19]. Nevertheless, a reduction of the visual acuity was still observed in 37.2% of eyes. Among them 4,5% became blind and 5.5% lost their useful vision. Obviously, the reduction of visual acuity was higher in eyes with posterior uveitis (11.1%) and retinal vasculitis (30.3%) [19]. It has been shown also that MTX is potent for anterior uveitis when used at a low dose of 7.5 to 25 mg weekly [20].

### 3.4. Cyclophosphamide

Cyclophosphamide is a nitrogen mustard-alkylating agent, the active metabolites of which are alkylate purines in DNA and RNA and result in cross-linking, aberrant base pairing, ring cleavage, and depurination [11]. In patients, it decreases the number of activated T lymphocytes, suppresses helper T lymphocytes functions, and decreases B-lymphocytes for months. Davatchi compared in a double blind controlled crossover study the short-term efficacy of pulse cyclophosphamide (PCP) plus prednisolone versus placebo over prednisolone alone. The mean visual acuity improved from 3.7 ± 3.2 to 4.9 ± 3.9 (*t* = 3.309, *P* < 0.002) in the PCP group and from 4.4 ± 3.6 to 4.5 ± 3.5 (*t* = 0.317, *P* = 0.75) in the placebo group. In the PCP group, VA improved in 57% of the eyes (95% CI: 44–60) and remained stable in 22% (95% CI: 11–33) but deteriorated in 21% (95% CI: 10–32). In the placebo group, 45% of the eyes improved (95% CI: 32–58), 14% remained stable (95% CI: 5–23), and 41% deteriorated (95% CI: 28–54). For this reason, the combination of PCP and prednisolone is superior compared to prednisolone alone in maintaining visual acuity [21]. Other parameters such as disease activity index improved more remarkably in the PCP group than in the placebo group, but differences were not statistically significant.

### 3.5. Biologic Agents

Looking at conventional treatment visual outcome, there are still high percentages (20–30%) of patients with a reduction of visual acuity and 10% of patients becoming blind from posterior eye involvement and uveitis complications.

In this scenario, biologic agents represent a valid alternative for patients nonresponsive to conventional treatments for achieving a stabilization of visual acuity and avoiding blindness. Interferon-alpha and TNF-alpha antagonists are the most frequently used biologic agents; recently, IL-1 blocking agents have been used with satisfactory results in terms of visual acuity maintenance.

Although the published literature consists of open and observational studies and while there are not yet available randomized controlled data on these drugs, the results available demonstrate a favourable response to biologic agents.

### 3.6. Interferon-Alpha

Interferons are a group of cytokines that include interferon-alpha-2a which is used to treat patients with severe ocular involvement and sight-threatening uveitis entities nonresponsive to conventional immunosuppressive agents. Although there is no controlled data, open and observational studies have shown the efficacy of interferon-alpha (IFN-alpha) in controlling uveitis attacks and reducing relapses [22–29]. However, there is no consensus about the ideal dose and duration of the treatment for ABD uveitis. For this reason, Onal investigated the long-term efficacy and safety of low-dose and dose-escalating therapy of IFN-alpha-2a in the treatment of uveitis in ABD.

This study included 37 patients receiving a daily dose of 3.0 million IU (MIU) subcutaneously for 14 days. Maintenance dose was achieved with 3.0 MIU 3 times per week given subcutaneously. The dosage was increased sequentially if uveitis relapses occurred. Total therapy duration was 24 months. Improvement in visual acuity was achieved in 41% of patients with doubling of the visual angle associated with a decreased rate of uveitis' relapses [22]. Similar results with low dose of IFN-alpha-2a were obtained by Guedry that described stabilization or at least an improvement of visual acuity in 87.5% of eyes at two years of treatment [23]. In 2010, Sobaci presented the results of his prospective study; patients were treated with INF-alpha-2a 4.5 MIU 3 times per week for the first 3 months, followed by INF-alpha-2a 3 MIU for the next three months. Visual acuity improved in 28.3% of eyes and was maintained in 76.7% during the follow-up period [24].

Kötter treated patients with an initial higher dose of IFN-alpha-2a starting with 6 MIU subcutaneously daily for at least 14 days and reducing it until discontinuation. Visual acuity improved in 75.3% (*n* = 55) of eyes, remained stable in 22% (*n* = 16) of eyes, and worsened in 2.7% (*n* = 2) of eyes. The increase of visual acuity for the right eyes was 0.33, and for the left eyes 0.36 logMAR. In 7 eyes, the final visual acuity was inferior to 0.1 logMAR but remained unchanged from the beginning [25, 26]. Kötter obtained a response rate of 92% in a relatively short time of 2 to 4 weeks, and discontinuation of the treatment was possible in 40% of patients. After 5 years of follow-up in patients treated with IFN 2-alpha, 67% of eyes obtained an increase of two lines or more in visual acuity [27]. Similar results were obtained by Deuter with an improvement or at least maintenance of visual acuity in 94.8% of eyes treated with a dose of 6 MIU subcutaneously daily for at least 14 days that is then tapered to a maintenance dosage of 3 million IU twice per week and finally discontinued, if possible. Median visual acuity was 0.30 logMAR at the beginning and improved to 0.07 logMAR at the end of follow-up period. Only 12.5% of eyes had a final visual acuity of 1.0 logMAR or less, due to preexisting irreversible ocular damage [28]. Tugal-Tutkun I. observed in her retrospective analysis between September 2001 and May 2005 similar results in terms of visual acuity recovery. As a matter of fact, the best visual acuity was achieved after a median of 4 months of IFN-alpha therapy and was found to be 0.28 ± 0.34 logMAR units in the right eye and 0.45 ± 0.56 logMAR units in the left eye. The best level of visual acuity achieved by IFN-alpha therapy was preserved throughout follow-up in 95% patients [29]. In her series of patients, only 36.4% of patients remained relapse-free, and complete remission was achieved only in 20% of patients compared with the data reported by Kötter where 82% of patients were relapse-free and 40% of patients achieved complete remission [26]. Patients treated with IFN-alpha often experienced a flu-like syndrome, weight loss, and often depression. For these reasons, the use of this drug is quite limited.

The main advantage of IFN-alpha treatment seems to be the possibility of discontinuation of treatment without relapse in at least 50% of patients and the preservation of visual acuity in almost 90% of patients compared with standard immunosuppressive therapy and even when compared with anti-TNF [30]. However, as assessed by Bodaghi and colleagues [31], the efficiency of IFN-alpha in sight-threatening uveitis seems to act more to suspend rather than to cure and for this reason it may be proposed as a second-line therapy after failure of conventional immunosuppressive treatments in nonresponsive patients. In this way, nonresponsive patients could avoid sight-threatening complications and blindness.

### 3.7. TNF-Alpha Antagonists

Tumor necrosis factor-alpha is a pleiotropic cytokine that has been shown to be elevated in patients with Behçet's disease and other autoimmune diseases. Numerous cells, also lymphocytes, produce TNF-alpha and their targets are two receptors known as p55 (TNF-R1) and p75 (TNF-R2). When TNF-alpha is produced during inflammation, it activates T-cells and macrophages and determines upregulation and expression of endothelial adhesion molecules and proinflammatory cytokines [32].

The first available molecule targeting TNF-alpha was a chimeric IgG monoclonal antibody infliximab (Remicade, Schering-Plough Pharma Inc.); later etanercept (Enbrel, White Pharmaceuticals Inc.), a p75 TNF-alpha receptor fusion protein, was developed. The last anti-TNF-alpha agent produced was adalimumab (Humira, Abbott Pharmaceuticals Inc.), a recombinant human IgG1 monoclonal antibody [32].

### 3.8. Infliximab

Infliximab infusion, used at a dose of 5 mg/kg every 6–8 weeks, is demonstrated to be effective in reducing ocular relapses and maintaining visual acuity [33–41]. Sfikakis described in a case series the effect of infliximab in 5 patients treated with standard immunosuppressive therapies. He observed a rapid and effective suppression of the ocular inflammation in these patients after seven days, confirmed also by an improvement and stabilization in visual acuity in all cases during the follow-up time [33]. These preliminary observations were confirmed also by Ohno and colleagues that described a significant reduction in the frequency of uveitis attacks during the efficacy-evaluation period of their study, and an improvement in visual acuity was noted in eyes in which uveitis remained in remission [34]. The visual acuity improvement obtained with the infusion of the anti-TNF-alpha agents could reach the sixth line of visual acuity as reported by Bodaghi and colleagues. This was a retrospective study of 12 patients (21 eyes) followed for a mean of 17.4 months (range: 8–30) [35]. Tugal-Tutkun described the long-term results of infliximab infusions of 5 mg/kg administered at weeks 0, 2, 6, and 14 and the patients were observed since enrolment for 54 weeks. The visual acuity improved significantly during the infusion period (weeks 0–22) but then decreased during the observational period (23–54 weeks). This was explained by the authors both for the frequency of uveitis attacks and for the need of corticosteroids treatment after the initial beneficial effects of infliximab infusions. For these reasons, treatment with anti-TNF-alpha agents should be continued in order to maintain the beneficial effects in cases of nonresponsive uveitis [36]. In a review paper in April 2007, Sfikakis et al. suggested that infliximab was the best available treatment in acute sight-threatening ocular inflammation in patients affected by Behçet's disease and should be used alone or as an add-on therapy in selected cases [37].

Compared with corticosteroids, high-dose methylprednisolone intravenously (1 g/day for 3 days), or intravitreal triamcinolone acetonide (4 mg) at the attack's onset, infliximab was equally effective in improving visual acuity from baseline but with less complications such as cataract or glaucoma and with a faster effect [38]. Yamada retrospectively compared the efficacy of infliximab versus CSA and described an improvement of visual acuity in 97% of eyes treated with infliximab versus the 93% of patients treated with CSA; the difference was not statistically significant. Nevertheless the study demonstrated that infliximab was more effective than CSA in the first 6 months [39]. Beside the rapid effect of action, a multicenter prospective study showed that uveoretinitis improved in 92% accompanied by visual acuity that improved from 0.736 logMAR at the first infliximab infusion to 0.616 logMAR after 1 year and remained unchanged in the other patients. Infliximab also decreased the frequency of recurrence and 44% of patients were attack-free after twelve months [40]. In addition, other studies demonstrated the efficacy of infliximab in improving visual acuity by reducing optic disc neovascularization and background retinal vascular leakage, as demonstrated with fluorescein angiography. The best corrected visual acuity was maintained or improved in 92.8% of eyes at 12 months, and in 80% of eyes at 24 months [41].

Finally we can conclude that almost all patients treated with infliximab, alone or as an add-on therapy, were nonresponsive to conventional treatments and achieved a fast suppression of the acute ocular inflammation. Less data is available on long-term outcomes, since it is also well known that the development of human antichimera antibodies (HACAs) has been implicated in the observed decline in therapeutic response to infliximab. The use of infliximab, as other anti-TNF-alpha agents, is not totally safe; Neri et al. in 2004 reported about the reactivation of tuberculosis under infliximab in a patient with ABD; remember that the endemic areas for ABD are also endemic for tuberculosis [32].

### 3.9. Etanercept

Etanercept is a recombinant human p75 TNF-alpha receptor artificial fusion protein; in a double-blind, placebo-controlled study of 40 male patients with ABD, Melikoglu et al. [42] reported that etanercept (25 mg twice/week, for 4 weeks) was effective in suppressing most mucocutaneous lesions in Behçet's patients. Etanercept was also given to treat children with ABD-associated uveitis at a dose of 0.4–0.5 mg/kg administered twice weekly as subcutaneous injections. At study entry, 39/42 eyes had active uveitis with normal visual acuity in 59% of eyes, impaired visual acuity in 10% of the eyes, and legal blindness in 31% of the eyes. Legal blindness was bilateral in 14% of the eyes. Ten of the 21 patients (48%), with 19 affected eyes, had normal best corrected visual acuity in both eyes. The causes of legal blindness were cataract, cystoid macular edema, and retinal detachment. Patients were treated with etanercept or infliximab, and at the end of the study best corrected visual acuity improved in 5/11 patients (7/16 eyes, 43%). Four patients (5/16 eyes, 31%) improved as a result of cataract surgery. In two eyes (two patients, both on infliximab), improvement of visual acuity from 20/400 to 20/70 and from 20/400 to 20/20, respectively, was unrelated to cataract surgery. Vision decreased in 2/18 patients (2/27 eyes, 7%) from 20/100 to 20/200 and from 20/60 to 20/200, respectively, both on etanercept (13% of etanercept treated eyes) due to uncontrolled inflammation, and remained unchanged in 16 patients (25 eyes) during the study (difference between etanercept and infliximab treated group, *P* = 0.48). The difference in improvement of visual acuity between etanercept and infliximab treated patients was not statistically significant; however, the authors concluded at the end of the paper that infliximab seemed superior to etanercept not only because of decreasing the number of concomitant medications required to control the uveitis, but also due to a lower rate of new-onset glaucoma and cataract [43].

In conclusion, we can consider the soluble TNF receptor as an alternative for nonresponsive patients to the other anti-TNF-alpha agents.

### 3.10. Adalimumab

Adalimumab is a recombinant human IgG1 monoclonal antibody targeting the TNF-*α*; it also binds soluble and the membrane-bound form of TNF-*α* [32]. Adalimumab, as previous biologic agents, has been used to treat nonresponsive ocular Behçet syndrome as it has some advantages: first of all, the self-administration and then a lower risk of anti-drug antibody formation. In 2007, there was a first case series describing the effect of adalimumab in sight-threatening uveitis; in all three patients treated, the visual acuity remained stable; and in two eyes of two different patients, an improvement also has been observed [44]. In 2010, Bawazeer et al. described the improvement of visual acuity and the corticosteroid and immunosuppressive sparing effect of adalimumab in 11 patients with ocular Behçet's disease. Adalimumab was administered subcutaneously at a dose of 40 mg every 2 weeks; of the 21 eyes, 17 had an improvement of visual acuity by 4.3 (range: 0–8) lines; 4 eyes of 3 patients did not improve in visual acuity [45]. Takase et al. reported their experience describing the successful switching from infliximab to adalimumab in ABD patients. Changing to adalimumab induced clinical remission again, thus suggesting that adalimumab can be an effective alternative to infliximab for patients having a hypersensitivity to this drug [46]. The application of adalimumab can also be hypothesized beside nonresponsive uveitis in panuveitis and pediatric uveitis [47, 48]. Unfortunately, adalimumab treatment is not totally safe, as multiple side effects have been reported recently such as reactivation of latent tuberculosis, endogenous endophthalmitis, retrobulbar neuritis, bilateral optic neuropathy, precipitation of systemic lupus erythematosus, secondary malignancies, lymphoma, and cardiac failures [32].

Although the results provided in ABD can be promising, no controlled trial is available and it is not possible to compare the characteristics of different anti-TNF-*α*, so that they are limited to nonresponsive cases of the disease.

### 3.11. IL-1 Blocking Agents

IL-1-blocking agents have started to be used recently in sight-threatening and nonresponsive cases. A small study on 7 patients treated with a single infusion of XOMA 052 (gevokizumab) 0.3 mg/kg demonstrated a rapid and meaningful improvement of the visual acuity starting from day 1, except in two patients; resolution of the ocular inflammation was observed in 5 patients at day 28; and all these 5 patients remained attack-free for a median of 49 days. XOMA 052 was well tolerated and no drug-related adverse events were observed [49]. Another recombinant form of human IL-1 receptor antagonist is anakinra, which is approved for the treatment of Rheumatoid Arthritis in combination with methotrexate. Emmi et al. described the use of this drug in a patient with ABD and serious ocular involvement. The patient was treated with anakinra at a dose of 100 mg/day and after three months from the beginning of the treatment, ocular inflammation had disappeared and visual acuity from 20/50 in RE and 20/32 in LE was restored to 20/20 in both eyes [50]. Ugurlu published the favourable results obtained with canakinumab, another IL-1 receptor antagonist, treating a 16-year-old female with ABD and ocular involvement nonresponsive to other immunosuppressives. The young patient treated with a single dose of 150 mg of canakinumab obtained a resolution of the ocular inflammation in the right eye with a visual acuity of 0.4. The left eye was not evaluated because of the presence of a complicated cataract. The patient remained free of attacks during the 8 weeks of follow-up. Interestingly, this patient was nonresponsive to anakinra but obtained a remission of the ocular inflammation with canakinumab. Both agents are IL-1 blockers but may act in different ways and have different half-lives. While anakinra blocks both IL-1*α* and IL-1*β*, canakinumab specifically targets IL-1*β*. It was also noticed that, after the infusion of IL-1 blocking agents, the circulating white blood cells developed a less inflammatory phenotype compared with baseline [51].

Although these studies have limitations resulting from their design and the small number of patients, the results do support additional studies to evaluate the role of IL-1 blocking agents for the treatment of uveitis and retinal vasculitis and for nonocular ABD manifestations as well as other types of noninfectious inflammatory uveitis.

## 4. Changes in the Course of the Disease Over Time

ABD is a rare disease but has a relatively high prevalence along the ancient “Silk Road.” Since 1950 until the present, the medical treatments options have been evolving to obtain a better control of the inflammation, a reduction of the sight-threatening complications, and a better final visual outcome.

The first drugs introduced to manage this syndrome were steroids, followed then by immunosuppressive agents (alkylating agents, nucleoside analogues, folic acid analogues, and nitrogen mustard-alkylating agents) and finally biologic agents (INF-*α* and TNF-*α* antagonists and IL-1 blocking agents).

In a large series from Turkey, including patients seen between 1980 and 1998, initial visual acuity was 0.1 or less in 647/1567 eyes (41.2%) [1]. A recent multicenter Turkish study conducted in 2004 showed that a lower percentage (21.7%) of eyes with Behçet's uveitis had initial visual acuity of 0.1 or less, suggesting a trend toward a milder disease [52]. Cingu et al., who compared patients who presented in the period 2000–2004 with those who had presented a decade earlier, have confirmed this tendency also. The authors found a better initial, potential, and 3-year visual acuity in patients from the 2000s period and none of the patients became legally blind during the 3 years of follow-up in this group. This may be explained by a milder disease in this period and/or more frequent use of immunomodulatory treatment before referral [53]. Disease severity and visual outcomes of patients with ABD uveitis over the decades have been changing significantly also in other countries. In Japan, Yoshida A. et al. described between the 1980s and 1990s an increase in the percentage of eyes with good visual acuity (≥20/30) and a significant reduction in eyes with poor visual acuity (≤20/200); and they justified this change as a result of improvements in environmental factors (e.g., putative microorganisms, lifestyles, and hygienic situations) [54]. Similar to trends reported from Japan, Khairallah and associates found that Tunisian patients with ABD uveitis who presented after 2001 had a better final visual acuity than those who presented before 2001. Only 3.4% of patients suffered from blindness and 12.5% suffered from unilateral blindness. The authors justify this trend due to the use of immunomodulatory drugs as first-line therapy after 2001 but a milder disease could not be excluded because initial visual acuity was also better in the more recent study period [55]. The study by Kump and associates in the United States has shown a similar trend in a nonendemic population; mean visual acuity in the 1990s group was significantly better than in the previous decades (*P* < 0.001 for the 1960s group and *P* = 0.019 for the 1980s group) and it seems that newer immunomodulating and biologic agents may offer an improved prognosis in patients with ABD [8].

In a recent paper, Taylor reported improved visual prognosis in patients with ABD who presented at two referral centres in England and in Australia between 2000 and 2010. They estimated the risk of visual loss to be 39% and the risk of severe visual loss to be 24% at 10 years. Male sex, unilateral disease, and left eye involvement increased the risks of severe visual loss at 5 and 10 years in this series of patients. Patients who were treated with anti-TNF-*α* were less likely to have severe visual loss, respectively, at 5 and 10 years. Taken together, these results suggest that adequate immunosuppression can reduce the risk of severe visual loss in patients with ocular ABD but that azathioprine is not effective enough to achieve this goal [56]. Krause and associates in Berlin analyzed retrospectively the data of 140 patients with ABD and found that the risk of losing useful vision was 21% [57], less than 75% of Benezra and Cohen's study [6] and 72% less than described in another study published in 1995 in Germany [58]. Mean and median Snellen visual acuities were reported to be 0.3 and 0.6 at baseline and 0.4 and 0.8 after 3 years, respectively [57]. In Switzerland, another nonendemic country, mean Snellen visual acuity was 0.74 at baseline and 0.79 after a mean follow-up of 5.7 years [59]. ABD appears to be less severe in this area, but in presence of ocular clinical and angiographic involvement, the authors decided to treat patients even if the visual acuity was good; and the final visual outcome justified this aggressive treatment. In a recent study from China in a single center, which included 437 Behçet's disease patients seen between 1995 and 2006, final visual acuity was less than 0.1 in 20.4% of eyes after a median follow-up of 4 years [60].

Muhaya and associates compared Behçet patients seen in Japan and the United Kingdom in a cross-sectional observational study conducted simultaneously at two centres. The duration of ocular disease was around 7 years in both cohorts. Even if the treatment schedules were very different and Japanese patients had more active disease, the visual results were comparable. Visual acuity was worse than 6/60 in 31% of patients in Japan and 21% of patients in the United Kingdom; oral steroids and azathioprine are widely used in London; on the contrary, colchicine was used instead in Japan. This cross-sectional study reveals differences in the clinical features and management of ocular disease in ABD patients, but this had no significant effect on the visual outcome at seven years [61].

## 5. Conclusion

In conclusion, in order to restore and maintain good visual acuity in ABD with ocular involvement, the importance of the early use of combined immunomodulatory regimens and use of biologic agents seems clear. This is much more effective in nonresponsive cases where the reduced severity and number of uveitis attacks can prevent early visual loss [62]. The evidence-based European League Against Rheumatism (EULAR) recommendations for the management of ABD, published in 2008 and still valid, suggest for posterior eye involvement a treatment regimen that includes systemic steroids and azathioprine [63]. Corticosteroids rapidly suppress the inflammation but potential side effects, including cataracts and glaucoma, cause concern. Azathioprine is widely accepted as the initial agent for ocular involvement of ABD. The EULAR committee discussed also a possible role of azathioprine as a prophylactic treatment in patients with ABD at high risk of developing eye involvement; but it was decided that more prospective data were needed.

In case of severe eye involvement, identified as a drop of two lines or more in visual acuity on a 10/10 scale and/or retinal disease (retinal vasculitis or macular involvement), another immunosuppressive needs to be added. Particularly, it is recommended that either cyclosporine A or TNF-alpha antagonists such as infliximab may be used in combination with azathioprine and corticosteroids; alternatively, INF-alpha with or without corticosteroids could be used.

Due caution for hypertension and nephrotoxicity is important when using cyclosporine A; instead reactivation of tuberculosis has to be considered when using TNF-alpha antagonists. IFN-alpha, alone or in combination with corticosteroids, appears to be a second choice in eye disease due to financial and safety concerns, mainly depression and cytopenias [63]. Recently, IL-1 blocking agents have been used in sight-threatening and nonresponsive cases with preliminary good results [49–51].

Another important issue to consider when using immunomodulatory drugs and biologic agents is the switching effect in the event of failure of desired response to one biologic therapy [64]; the efficacy of changing from a biologic agent to another one suggests changing the drugs either for poor response (primary failure) or for progressive decrease of efficacy because of the production of patient antibody reaction to the nonhuman part of the chimeric molecule used for treatment (secondary failure).
